# Successful omental flap coverage repair of a rectovaginal fistula after low anterior resection: a case report

**DOI:** 10.1186/s40792-023-01642-7

**Published:** 2023-04-18

**Authors:** Yuta Kuhara, Hiroshi Hotei, Tatsunori Hashimoto, Shingo Seo, Ai Amioka, Naoki Murao, Aki Kuwada, Akira Nakashima, Ryutaro Sakabe, Kou Tahara

**Affiliations:** Department of Surgery, Kure Kyosai Hospital, Nishityuo-2-3-28, Kure, Hiroshima 730-0802 Japan

**Keywords:** Rectovaginal fistula, Rectal cancer, Low anterior resection, Omental flap, Muscle flap

## Abstract

**Background:**

Rectovaginal fistula (RVF) is a troublesome and refractory complication after low anterior resection (LAR) for rectal cancer. An omental flap repair was performed for the RVF caused due to Crohn’s disease and childbirth trauma. However, there are few cases of an omental flap repair for RVF after LAR. Herein, we present a successfully repaired case of RVF by omental flap coverage after LAR for rectal cancer.

**Case presentation:**

A 50-year-old female patient with advanced rectal cancer underwent laparoscopic LAR with double-stapling technique anastomosis and achieved curative resection. She complained of a stool from the vagina and was diagnosed with RVF on the postoperative day (POD) 18. Conservative therapy was ineffective. We performed laparoscopic fistula resection and direct closure of the vagina and rectum, designed the omentum that could reach the pelvis, repaired RVF by omental flap coverage, and performed transverse colostomy on POD 25. She was discharged on initial POD 48. Seven months after the initial operation, colostomy closure was administered. There was no recurrence of RVF found 1 year after the initial operation.

**Conclusions:**

The patient achieved an omental flap coverage for RVF. We successfully performed the omental flap coverage repair in patients with RVF after the leakage of LAR. An omental flap may become an alternative treatment for muscle flap or an effective treatment for RVF.

## Background

Rectovaginal fistula (RVF) is a troublesome and refractory complication. RVF was caused due to Crohn’s disease, childbirth trauma, hysterectomy, and low anterior resection (LAR), etc. [1]. Although various surgical procedures, for example, ileostomy, colostomy, perineal approach, muscle flap transposition, and omental flap coverage have been reported [1–3], RVF is unaltered or may not be cured. A few cases of omental flap coverage repair after LAR have been reported [2, 4], but the cause of RVF was not leakage after LAR. Herein, we report a successful repair of omental flap for RVF because of the leakage after LAR.

## Case presentation

**Fig. 1:**
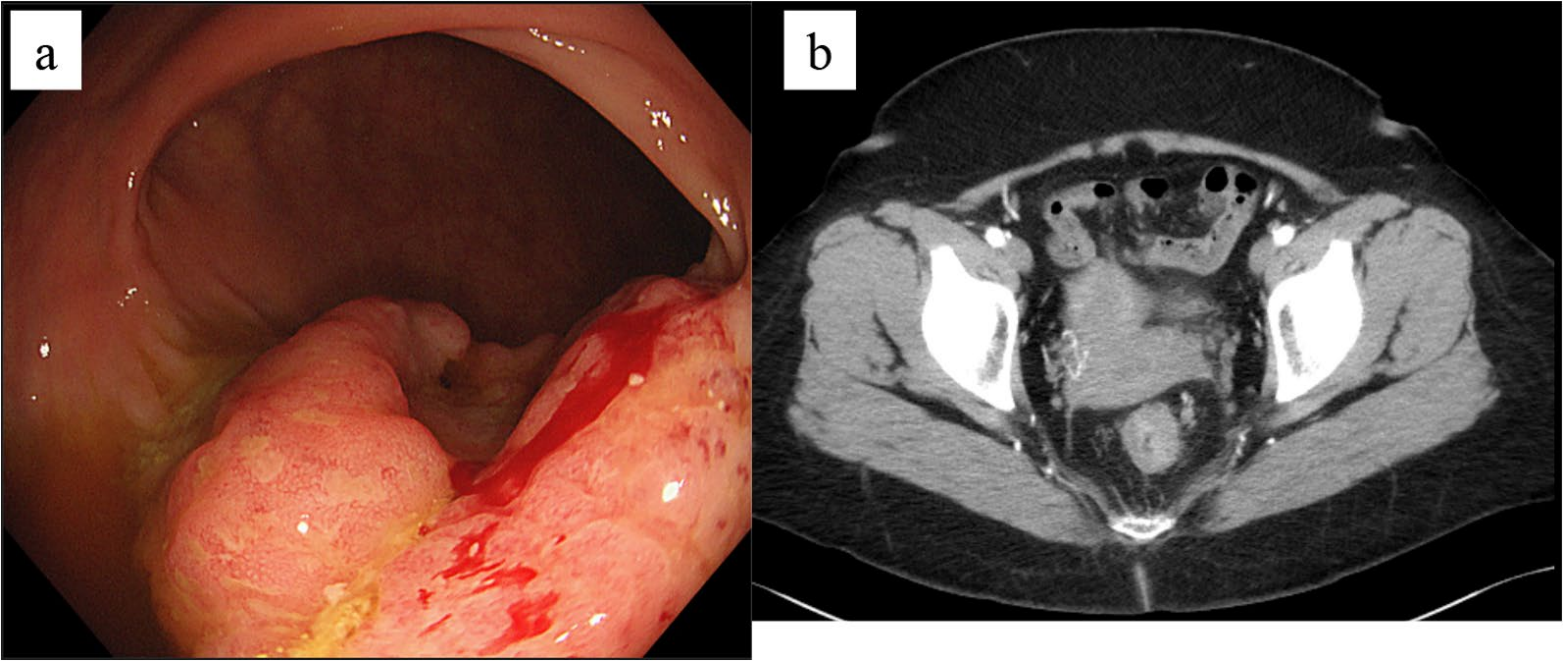
**A** Colonography revealed an advanced rectal cancer. **B** Computed tomography scan revealed a hypervascular irregular wall thickness in the rectum and swollen lymph node

**Fig. 2:**
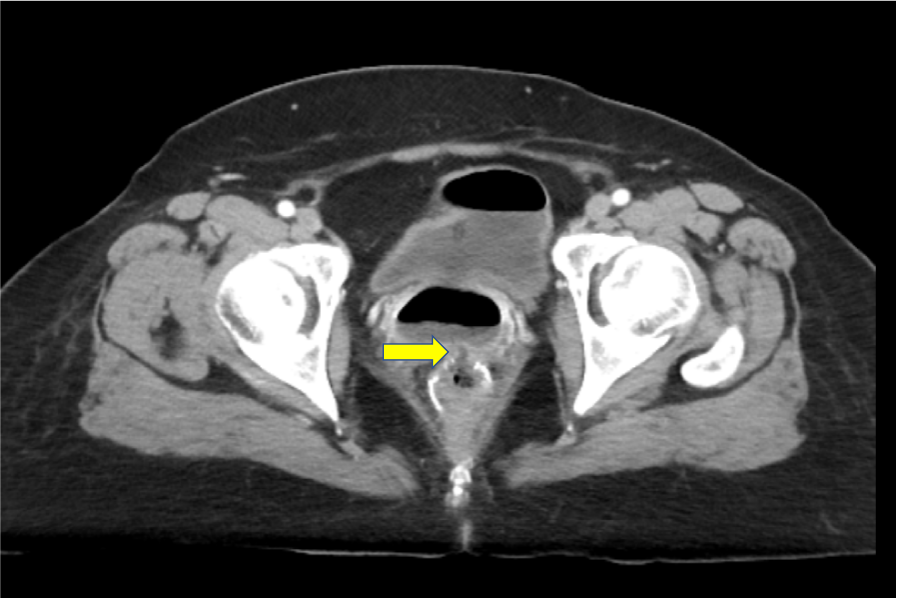
Computed tomography scan revealed a fistula between the vagina and the rectum (arrows)

**Fig. 3:**
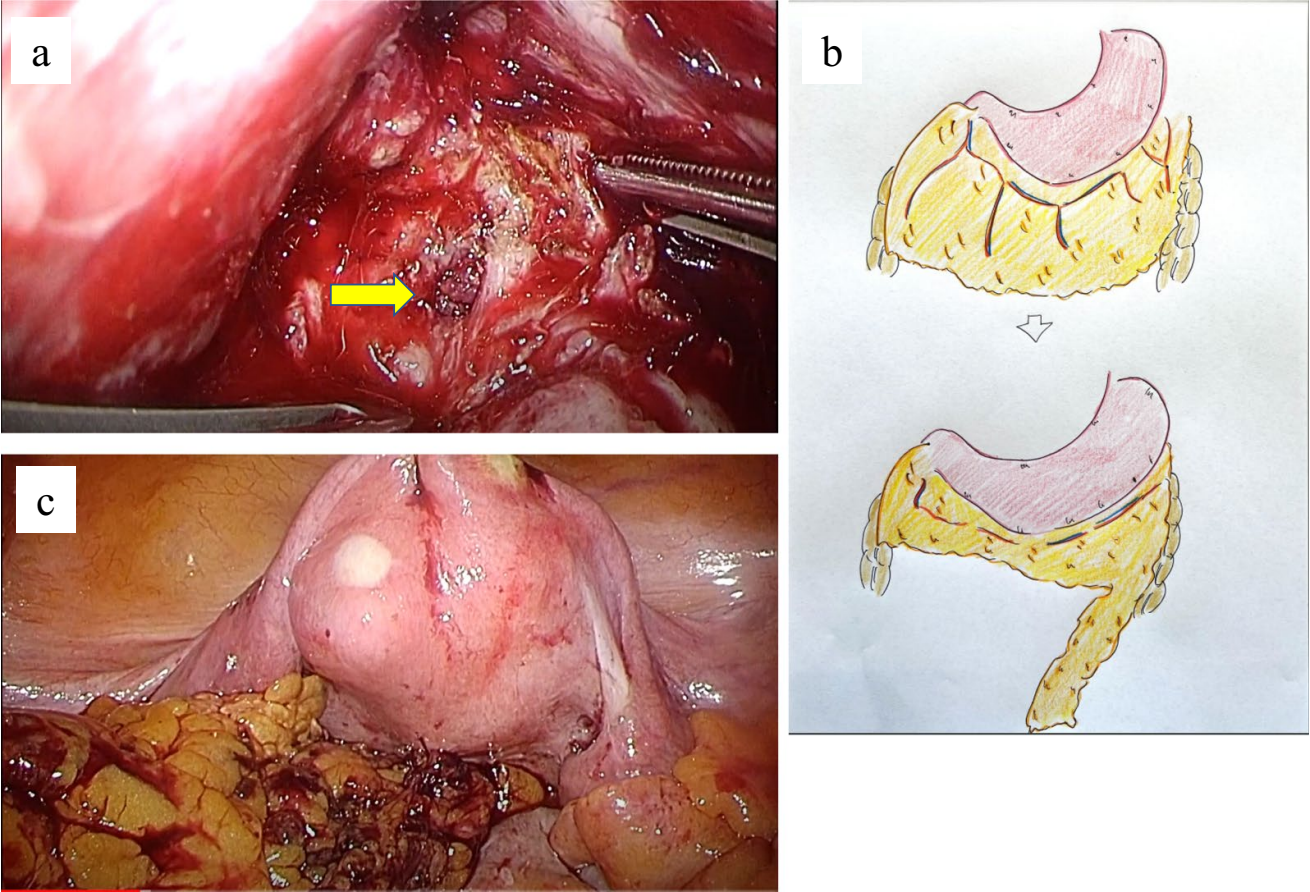
**A** The leakage point in the rectum was sutured after resecting the fistula (arrow). **B** We designed the omentum which could reach the pelvis. **C** After covering the vagina and the rectum with the omentum flap

A 50-year-old female patient was referred to our hospital because of bloody stool. Colonography showed advanced rectal cancer and computed tomography (CT) scan revealed a rectal mass and a swollen regional lymph node (Fig. 1A, B). She underwent laparoscopic low anterior resection (LAR) with double-stapling technique anastomosis and achieved curative resection. She started to have liquid on POD 1 and a daily diet on POD 7. The bloody stool was found on POD 17 and she complained of stools and gas from the vagina on POD 18. CT revealed air in the vagina from the anastomotic part, and she was diagnosed with RVF (Fig. 2). Conservative therapy, with nothing by mouth and total parenteral nutrition, had been performed for 1 week. However, the treatment was unsuccessful. Therefore, we performed laparoscopic fistula resection and direct closure of the part of rectal leakage on POD 25 (Fig. 3A). We designed the omentum that could reach the pelvis and cover between the vagina and rectum (Fig. 3B). In addition, we fixed omentum flap with the uterus back side close to the vagina (Fig. 3C). We performed RVF repair by omental flap coverage. Then, we performed transverse colostomy. The operative time was 425 min and with a blood loss of 300 mL. Liquid food was started on POD 27 (re-operation POD 2), and daily food was started on POD 31 (re-operation POD 6). She was discharged on POD 48 (re-operation POD 20).

**Fig. 4:**
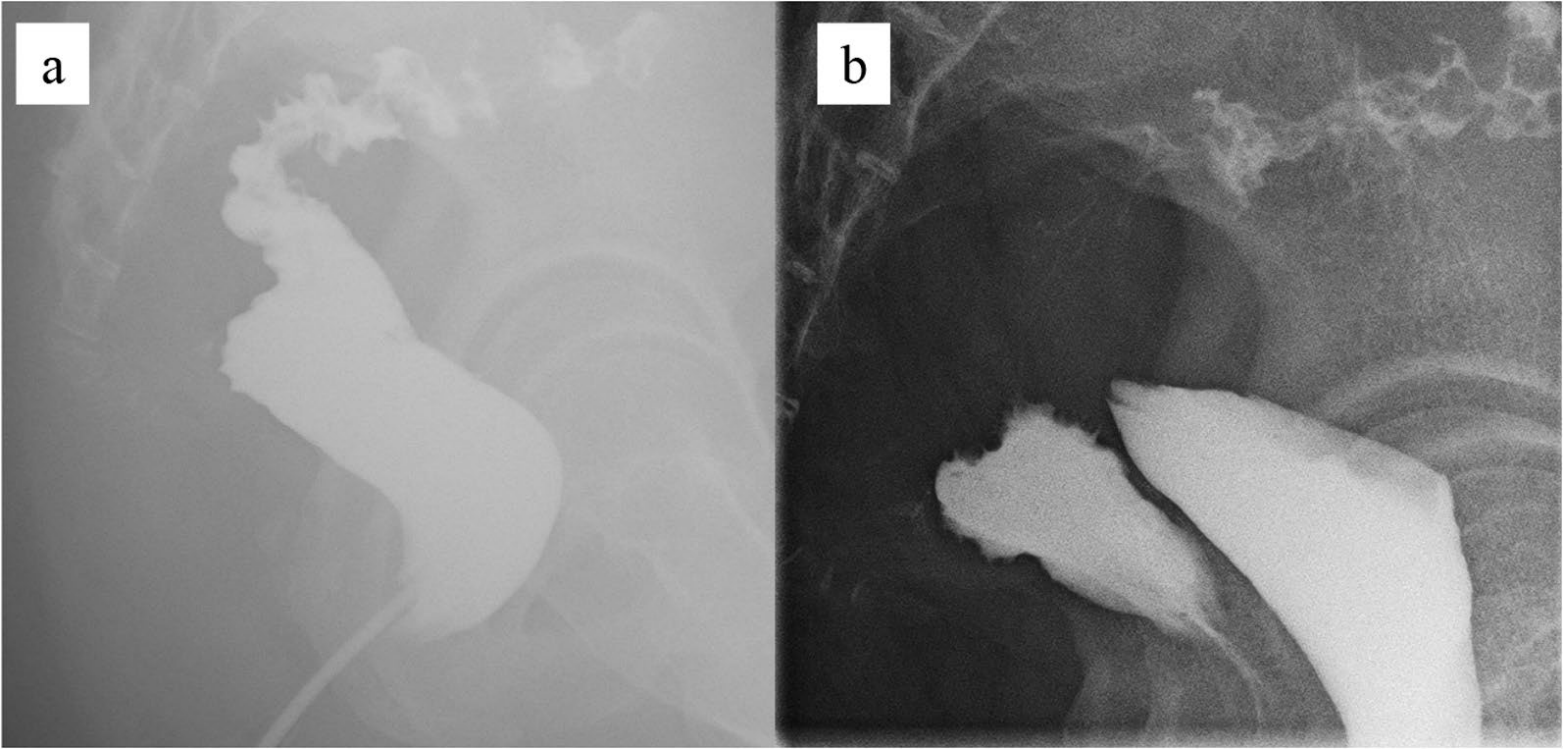
**A** Gastrografin enema from the anus. **B** Gastrografin enema from the vagina. These demonstrated no fistula between the vagina and the rectum

The pathological diagnosis was T3N1M0 stage IIIb (TNM classification) moderate adenocarcinoma. Adjuvant chemotherapy with capecitabine and oxaliplatin was administered for 6 months. Thereafter, the enema and transvaginal examination revealed no RVF (Fig. 4). Colostomy closure was performed 7 months after the initial operation. There was no recurrence of RVF found 1 year after the initial operation.

## Discussion

Anastomotic leakage is a critical complication in colorectal operations. Particularly, RVF is a type of anastomotic leakage after LAR, but RVF is worse complication than normal leakage [5]. The incidence of RVF ranges from 0.3 to 6% [6]. Another study reported an incidence of 9.7–12.4% [7, 8]. Prognostic nutritional index (< 45), preoperative chemotherapy, tumor size (≥ 50 mm), intraoperative bleeding, and lateral lymph node dissection are the risk factors for RVF after LAR as revealed by Watanabe et al. [9]. The other risk is the double-stapling technique caused unintentional incorporation of the vaginal posterior wall into the colorectal anastomosis anterior wall [10]. The patient had no risk factor and the vaginal posterior wall was not involved. Minor anastomotic leakage existed and damaged the vagina, in this case, thereby causing RVF.

RVF treatment is more difficult than the other complications, for example leakage. Conservative treatment is usually ineffective, especially considering that the diameter of the RVF with spontaneous healing was not larger than 1 cm [11]. Therefore, surgical treatment is often performed for RVF, such as colostomy or ileostomy for fecal diversion, fistula resection and direct closure, re-anastomosis, and muscle flap transposition, omental flap coverage. Omental flap repair for RVF caused by Crohn’s disease, childbirth trauma, and hysterectomy was reported [1]. However, only two cases of omental flap coverage repair after LAR have been reported [2, 4]. One case was an adjuvant radiotherapy case. The RVF was closed by directed sutures after transrectal and transvaginal excision of the fistula ostium, and transrectal omental flap and vaginal flap was performed. The RVF recurred on the postoperative day 8 [4]. The other case had the RVF caused by a megarectum. The rectovaginal septum was dissected up to the fistula tract and the openings on both vaginal and rectal sides were closed. The omental flap was interpositioned into the rectovaginal septum and pulled down by the perineal operator and secured at skin level. The patient showed no recurrence [2]. Although two cases of RVF were repaired by omental flap coverage, the cause of RVF was not leakage.

Only the fistula resection and the direct suture repair of the rectum and the vagina are highly probable recurrences. Several studies reported that the tissue interposition-like muscle, such as gluteal or gracilis muscles, is effective for RVF repair [12, 13]. Hence, the omental flap coverage was chosen for the tissue interposition. This is because the omental flap is less invasive than the muscle flap, surgeons of gastroenterological surgery are more familiar with the omental flap than the muscle flap, and there were no statistically significant differences found between the omental flap and the muscle flap in terms of mortality and sepsis [14]. We trimmed the omentum and filled up the omental flap between the rectum and the vagina after RVF resection and direct closure (Fig. 3).

Although omental flap coverage repair may be effective for RVF after LAR, it is possible that the designed omentum does not reach the pelvis, especially after the operation of the gastric cancer. Except for omentum resection case, if the omentum is designed from hepatic flexure to gastrosplenic ligament or splenic flexure, the omentum flap can reach the pelvic in most of the cases. If the designed omentum is unable to reach the pelvis, the designed omentum is made thin or the branches of the right gastroepiploic artery are resected [4]. We reach the omental flap of the pelvis along the descending colon to prevent internal hernia. The incidence of internal hernia after omental flap repair is not reported yet [1, 2, 4].

## Conclusions

Herein, we present a rare case of RVF repair after LAR. The patient achieved omental flap coverage treatment for RVF. RVF treatment remains refractory. However, we successfully performed an omental flap coverage repair in a patient with RVF after the leakage of LAR. An omental flap may become an alternative treatment for muscle flap or an effective RVF treatment after LAR.

## Data Availability

All data generated or analyzed during this study are included in this published article.

## Abbreviations

RVF: Rectovaginal fistula
LAR: Low anterior resection
POD: Post-operative day
CT: Computed tomography
